# Family member information extraction via neural sequence labeling models with different tag schemes

**DOI:** 10.1186/s12911-019-0996-4

**Published:** 2019-12-27

**Authors:** Hong-Jie Dai

**Affiliations:** 1 Department of Electrical Engineering, National Kaohsiung University of Science and Technology, Kaohsiung, Taiwan, Republic of China; 2 School of Post-Baccalaureate Medicine, Kaohsiung Medical University, Kaohsiung, Taiwan, Republic of China; 3 Interdisciplinary Program of Green and Information Technology, National Taitung University, Taitung, Taiwan, Republic of China

**Keywords:** Family history information extraction, Named entity recognition, Neural sequence labeling modeling

## Abstract

**Background:**

Family history information (FHI) described in unstructured electronic health records (EHRs) is a valuable information source for patient care and scientific researches. Since FHI is usually described in the format of free text, the entire process of FHI extraction consists of various steps including section segmentation, family member and clinical observation extraction, and relation discovery between the extracted members and their observations. The extraction step involves the recognition of FHI concepts along with their properties such as the family side attribute of the family member concept.

**Methods:**

This study focuses on the extraction step and formulates it as a sequence labeling problem. We employed a neural sequence labeling model along with different tag schemes to distinguish family members and their observations. Corresponding to different tag schemes, the identified entities were aggregated and processed by different algorithms to determine the required properties.

**Results:**

We studied the effectiveness of encoding required properties in the tag schemes by evaluating their performance on the dataset released by the BioCreative/OHNLP challenge 2018. It was observed that the proposed side scheme along with the developed features and neural network architecture can achieve an overall F1-score of 0.849 on the test set, which ranked second in the FHI entity recognition subtask.

**Conclusions:**

By comparing with the performance of conditional random fields models, the developed neural network-based models performed significantly better. However, our error analysis revealed two challenging issues of the current approach. One is that some properties required cross-sentence inferences. The other is that the current model is not able to distinguish between the narratives describing the family members of the patient and those specifying the relatives of the patient’s family members.

## Background

Family history information (FHI) is known to be essential for understanding disease susceptibility and is critical for individualized disease prevention, diagnosis, and treatment [1, 2]. Many care process models relied on family history information in their decision-making process of diagnosis and treatment. For example, Y Wang, L Wang, M Rastegar-Mojarad, S Liu, F Shen and H Liu [3] demonstrated the potential use of family history information in predicting medical problems. In order to provide a comprehensive patient-provided FHI to physicians, there is a need to develop natural language processing (NLP) systems that are able to automatically extract such information from electronic health records (EHRs).

The extraction of FHI from unstructured EHRs consists of various steps [4]: 1) Section segmentation: a preprocessing step to identify the sections containing FHI; 2) Family member and clinical observation extraction: a fundamental step to recognize family member mentions and their potential clinical observations described in the corresponding sections; 3) Family member-observation relation discovery: The final step associates the extracted observations with the correct family members. The established FHI extraction systems can then be applied to develop methods to aid clinical decision support, assess the risks of cancers, identify family pedigrees and foster downstream analyses as presented by previous studies [4, 5].

**Fig. 1:**
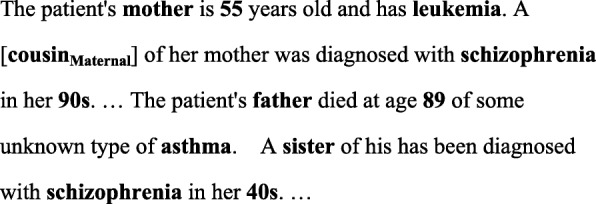
An example of the annotation of the family history information extraction task

To standardize the evaluation protocol of FHI extraction, the BioCreative/OHNLP challenge 2018 [6] released a corpus annotated with FHI. Figure 1 shows an example of the annotations in the released FHI extraction (FHIE) corpus. The annotations include: 1) Family members and the attributes of their family side (e.g. the “Maternal” annotation for “cousin”). For first degree relatives (i.e. the “mother” and “father” in Fig. 1), the side of family is “NA”; 2) Clinical observations of health-related problems including diseases, smoking, suicide, and drinking; 3) The age and the living status related to the family members (not shown in Fig. 1). In addition, the corpus only contains clinical texts extracted from the family history section of the EHRs. The goal of the BioCreative/OHNLP entity recognition subtask is to develop a system that can provide a document-level list of family members along with their family side attributes and clinical observations described in the EHRs. Furthermore, all extracted family member mentions must be normalized to their corresponding names listed in Table 1.

**Table 1:** The Normalized Family Names

Degree	Normalized Family Names
1	Father, Mother, Parent, Sister, Brother, Daughter, Son, Child
2	Grandmother, Grandfather, Grandparent, Cousin, Sibling, Aunt, Uncle

The FHIE problem can be formulated as a classification task in which several classifiers were developed for the target concepts. In addition, it can be formulated as a sequential labelling task by first identifying target concepts and the corresponding attributes, and then aggregating them to build the list. In this study, we followed the second approach and constructed a neural sequential labeling model to address the FHIE problem. The performances of the proposed methods were examined and analyzed on the FHIE corpus.

Specific contributions of this work are as follows:

- We designed three specific tag schemes for the task of family member and side attribute extraction along with corresponding methods to normalize the recognized family member terms and determine their properties.
- We demonstrated that the proposed side scheme is the most suitable tag scheme for the current released dataset because the family side information cannot be easily determined by rules. On the other hand, the relation-side scheme has the potential for improvement due to its capability in distinguishing the relationships of second-degree relatives from those of the first-degree relatives.
- We exhibited the performances of different word embedding strategies and empirically showed that the pre-trained GloVe [7] provides better representation than the others for the FHIE problem.
- We explored the effectiveness of incorporating features based on the UMLS (Unified Medical Language System) [8] for clinical observation recognition and noticed that the inclusion of UMLS embedding assisted the model in recognizing unseen observations.

## Methods

**Fig. 2:**
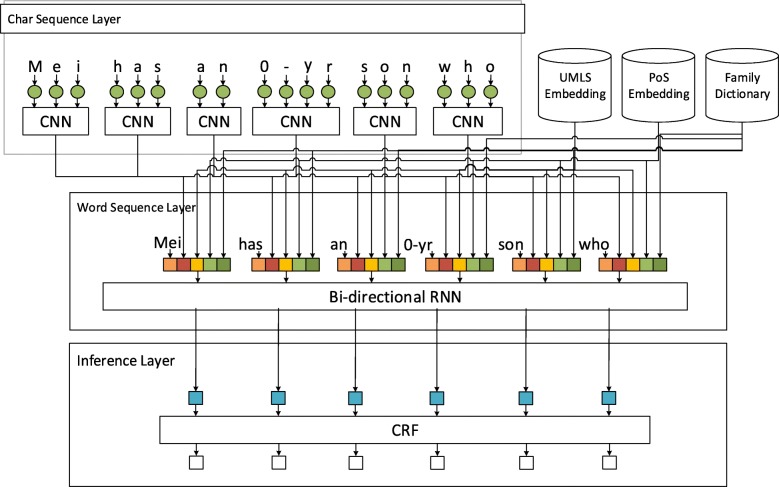
The neural sequence labeling model employed in the study for the family history information extraction task. Input containing numerical character such as “**0**-yr” was normalized to “0”

The patient note was preprocessed by our clinical toolkit [9] to segment sentences and generate the tokens and corresponding part-of-speech (PoS) information based on MedPost [10]. The numerical normalization method proposed in our previous work [11] was employed to normalize variations in the numerical parts of each token. We then linked the annotations of the gold standard to the generated sentences according to the span information provided in the FHIE corpus. All sentences including those that did not contain any family member or observation annotations were included in our training set to train the neural sequence labeling network model illustrated in Fig. 2. The network was based on the architecture proposed by X Ma and E Hovy [12]. The input of the network is the preprocessed sentences from an EHR with the output being the sequence of labels for tokens in the sentences. The implementation of the network will be elaborated later in the “Model Design” subsection.

### Tag scheme design

Since we formulated the task as a sequential labeling problem, one of the challenges is that we need to normalize the recognized family member mentions to one of the family member types listed in Table 1. In this study, we designed three IOB2-based tag schemes along with corresponding methods to normalize the recognized family member terms and determine their properties. Note that the three tag schemes were specifically designed for the family member concepts. For the observation concepts, we used the standard IOB2 scheme (B/I-Observation). The notation *fm* was first defined as the family member property, whose value is one of the strings listed in Table 1. The notation *sf* indicates the “side of family” property of each family member, which includes three possible values: NA (not available), Maternal and Paternal.

1. Standard scheme: In this scheme, we ignored the values of the *fm* and *sf* properties of each family member and represented all family member instances by using the “FamilyMember” tag. Therefore, five tags including B/I-FM, B/I-Ob and O were used. This configuration will be referred to as the baseline configuration hereinafter. The main advantage of employing the scheme is that the cost of the training phase is low, while the disadvantage is that we need to develop a post-processing algorithm to determine the type of family member and the property of the side of family.

**Fig. 3:**
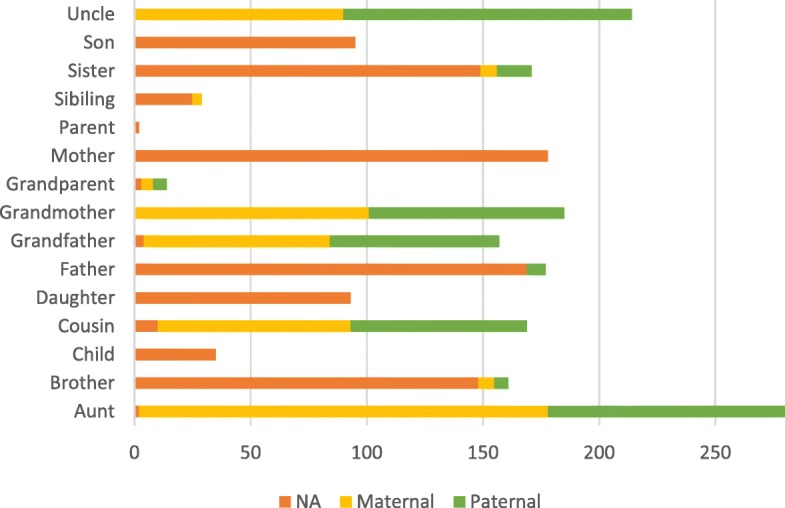
The distribution of the family members and the corresponding family side attributes in the training set of the FHIE corpus

We implemented a rule-based algorithm to determine these two values. The algorithm works by first identifying the value of the *fm* property. It removes adjective terms from the recognized mention and then transforms the remaining terms into their base forms for matching with the family names listed in Table 1. The algorithm also considers term variations like mom and mommy for “Mother”. The mention’s *fm* property is then set to the corresponding property of the matched value. On the other hand, for the *sf* property, the algorithm first checks whether the recognized mention follows a *sf* term. If it follows a *sf* term, then the corresponding *sf* value will be set. Otherwise, the *sf* value with the most number of occurrences for the family type of that mention will be set. Figure 3 shows the distribution of the family side attributes observed in the training set of the FHIE corpus. Take the term “cousin” in Fig. 1 as an example. The mention did not follow a *sf* term like maternal or paternal, so the algorithm will asign “maternal” as its family side attribute because the value appeared more frequently than “NA” or “paternal” in the training set.

2) Side scheme: In this scheme, the *sf* property was encoded in the tag set for family members. In our implementation, we relied on annotations that exist in the training set to determine the encoding in the tag scheme. For example, family members like “Mother” and “Daughter” shown in Fig. 3 were not associated with any family side values, so they were assigned with the B/I-FM_NA tag. The tag sets for other members include B/I-FM_SIDE_NA, B/I-FM_SIDE_Paternal and B/I-FM_SIDE_Maternal. The *sf* property was therefore determined based on the predicted tag for a recognized family member mention. The same algorithm designed for the baseline configuration was employed to determine the value of the *fm* property.
3) Relation-side scheme: In this scheme, both the *sf* and *fm* properties were encoded in the tag set for family members. Consequently, all possible combinations of the two properties that appeared in the training set were represented by the tag scheme. Take the family mention “cousin” in Fig. 1 as an example. The mention can be encoded as B-FM, B-FM_SIDE_Maternal, and B-FM_Cousin_Maternal in correspondence to the three tag schemes, respectively. The advantage of using this scheme is that we do not need to apply the post-processing algorithm designed for the other two schemes because the assignd tag itself provides sufficient property information.

### Model design

As shown in Fig. 2, our model consists of three layers: the character sequence representation layer, the word sequence representation layer, and the inference layer.

1. Character sequence representation layer: In this layer, we employed the character-level representation convolutional neural network (CNN) architecture with the max-pooling to capture the morphological information such as the prefix or suffix of a word. In accordinance with the CNN architecture proposed by X Ma and E Hovy [12], a dropout layer was applied before the character embedding was fed into CNN.
2. Word sequence representation layer: For word sequence representation, we used the pre-trained word embedding released by B Chiu, G Crichton, A Korhonen and S Pyysalo [13]. The embedding with the size of 200 dimensions was trained by applying the skip-gram model implemented in word2vec with a context window of 30 on a corpus collected from PubMed.

In Ma and Hovy’s model, the word embedding vector was concatenated with the character-level representation embedding to form the input vector of the bi-directional recurrent neural network (RNN). Herein the long short-term memory (LSTM) network was utilized to implement the bi-directional RNN layer. Furthermore, we augmented the input vector of the RNN layer by including the following three handcrafted features for each word. In our implementation, the concatenated vector was updated during training and regularized by adding a dropout layer before entering into the LSTM layer to improve the generalization of the model.

- PoS embedding feature: The PoS information is represented by a vector with a dimenstion of 20 randomly initialized from a uniform distribution.
- Family dictionary feature: This feature is a binary flag indicating whether the current word is a term referring to a family member.
- UMLS embedding feature: We included this feature to improve the ability of our model to capture observation concepts since UMLS is a comprehensive medical vocabulary that covers terms related to clinical observations. The semantic types listed in Table 2 were included in the recognition process. We exploited MetaMap [14] to recognize UMLS concepts mentioned in EHRs and the corresponding concept unique identifiers (CUIs) were extracted as features for the corresponding words. The recognized CUIs were represented by a 200-dimension concept vector trained by a skip-gram neural language model. The pre-trained concept embedding generated by L De Vine, G Zuccon, B Koopman, L Sitbon and P Bruza [15] was used to capture the semantic similarity between the concepts.

3) Inference layer: The output of the RNN layer becomes the input of the inference layer in which the conditional random field (CRF) was used to model the dependencies between labels in neighborhoods to jointly decode the best chain of labels for the given word representation sequence.

**Table 2:** Semantic Types Considered in This Study

Semantic Types (Abbreviation^a^)	
aapp, acab, aggp, anab, bacs, bdsu, bdsy, bird, blor, bpoc, bsoj, cell, cgab, clna, cnce, comd, drdd, dsyn, elii, emod, euka, famg, fndg, fngs, ftcn, genf, gngm, hlca, hops, idcn, inbe, inch, inpo, inpr, irda, lang, mamm, menp, mnob, mobd, neop, npop, orch, orga, orgf, patf, phsf, phsu, plnt, podg, popg, qlco, qnco, sosy, spco, tisu, tmco, topp, virs, vita	

^a^The full name definition can be found at https://mmtx.nlm.nih.gov/MMTx/semanticTypes.shtml.

For the layer with pre-trained embeddings, we applied a dynamic configuration in which the pre-trained vectors were fine-tuned with backpropagation. Details of the hyper-parameters used in this study are provided in Table 3. Most of the parameter settings follow the suggestion given by X Ma and E Hovy [12] except the embedding sizes for the proposed handcrafted features.

**Table 3:** Hyper-parameters of the Developed Neual Sequence Labeling Nework

Parameter	Value	Parameter	Value
word embedding size	200	Learning rate (LR)	0.01
char embedding size	30	Batch size	10
char embedding kernel size	3	Optimizer	SGD
number of char embedding kernels	50	Dropout	0.5
PoS embedding size	20	LR decay	0.05
UMLS embedding size	200	L2 regularization	1e-8
Epoch	1000		

### The corpus and evaluation metrics

The corpus released by the BioCreative/OHNLP 2018 challenge [6] was used to evaluate the developed models. The training set of the corpus consisted of 99 unstructured patient notes randomly sampled from the Mayo Clinic Employee and Community Health. 802 family members along with 978 observations were annotated.

The official document-level evaluation script released by the organizers was used to report the performance of the developed methods with different tag schemes. The script evaluated the normalized family members, their family side attributes and the observations extracted by our systems with the gold annotations by using the following metrics:

$$ \mathrm{Precision}\ \left(\mathrm{P}\right)=\raisebox{1ex}{$ TP$}\!\left/ \!\raisebox{-1ex}{$ TP+ FP$}\right. $$

$$ \mathrm{Recall}\ \left(\mathrm{R}\right)=\raisebox{1ex}{$ TP$}\!\left/ \!\raisebox{-1ex}{$ TP+ FN$}\right. $$

$$ {\mathrm{F}}_1=\raisebox{1ex}{$2\times P\times R$}\!\left/ \!\raisebox{-1ex}{$\left(P+R\right)$}\right. $$

where true positive (TP) denotes the number of correct predictions, false positive (FP) denotes the number of system outputs that do not exist in the gold standard annotations, and false negative (FN) denotes the number of gold standard annotations that do not exist in the system predictions. Both the values of the *fm* and *sf* properties that matched with the gold standard annotations can be counted as a TP. For the evaluation of the observations, a partial matching criterion was used which allows four mismatched tokens at most to determine the matching. For instance, the extraction of either “diabetes” or “type 2 diabetes” from the phrase “type 2 diabetes” will be considered as a TP.

## Results

### Results on the training sets

During the participation of the challenge, we applied a ten-fold cross validation (CV) to study the performance of the proposed method on the training set. The results are displayed in Table 4. All of the configurations were based on the same network architecture, features and the same hyper-parameters depicted in Table 3, with the only difference being the employed tag scheme as described earlier in the Methods section. All neural network architectures were implemented by using CUDA 8.0 and PyTorch libraries and trained on three machines equipped with the Nvidia GTX-1080ti graphics card. The results indicate that the model with the side-scheme achieved the best F-score and precision, while the model with the standard scheme had a better recall. The relation-side configuration obtained the lowest recall and F-score.

**Table 4:** Performance Comparison with CRF-based Methods on the Training Set with 10-fold Cross Validation

Configuration	Precision	Recall	F1-score
Baseline	0.882	0.857	0.870^a^
CRF-Baseline	0.836	0.743	0.787
Side	0.902	0.855	0.878^a^
CRF-Side	0.865	0.753	0.805
Relation-side	0.883	0.854	0.869^a^
CRF-Relation-side	0.850	0.700	0.768

^a^ Indicates passing the significant test under the level of 0.001. The *p*-values for the three configurations are 0.000006, 0.00005, and 0.000000004 respectively

We further conducted another ten-fold CV after the challenge and implemented three other models based on the CRF [16] with the designed tag schemes. The same algorithms developed for the neural models were also applied for the CRF models. Based on the results in Table 4, we observed that the PRF-scores of the CRF models showed a similar trend to the neural network-based implementations but with a significant lower performance.

### Results on the test sets

**Fig. 4:**
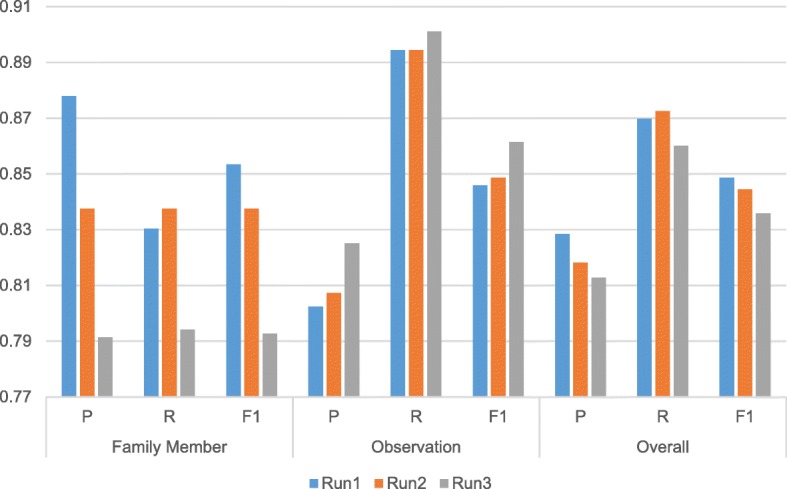
The official test set results for the family information

We submitted three runs for the FHIE entity recognition subtask with each corresponding to the model with side scheme (Run 1), the model with relation-side scheme (Run 2) and the baseline model (Run 3), respectively. The official results of the submitted runs on the test set are illustrated in Fig. 4. We can see that the model with the side scheme achieved the best overall F-score and the best F-score for family members. The baseline model had the worst overall and family member F-score which may be owing to the different family type distribution in the test set.

## Discussion

### Result comparison with the top-ranked teams

Table 5 demonstrates the performance of the three other top-ranked systems on the FHIE test set. The F-score of our best run ranked second in the challenge. X Shi, D Jiang, Y Huang, X Wang, Q Chen, J Yan and B Tang [17] achieved the best F-score by developing a joint learning model that can mutually determine the FH information and observations as well as the relations among them. Compared to our sequential labelling model for recognizing FHI concepts, their model was relatively simpler, which was a bidirectional LSTM-CRF network with only word and PoS embedding input layers. They utilized the pre-trained word embedding based on GloVe and the one-hot encoding to represent the PoS of each word generated by the natural language toolkit. The outputs of the bidirectional LSTM layer and the CRF layer were set as the input of another bidirectional LSTM to determine whether the recognized entity pairs have any relations. The loss function of the joint model was set as the cross-entropy function that consisted of entity recognition and relation extraction. Similar to our baseline configuration, they relied on post-processing rules which check the surrounding text for keywords such as “maternal” and “paternal” to generate the list of normalized family member names along with their family side attributes.

**Table 5:** The Performance of the Top-ranked Systems in the Family History Information Extraction Task

Team	Precision	Recall	F_1_-score
X Shi, D Jiang, Y Huang, X Wang, Q Chen, J Yan and B Tang [17]	0.8886	0.8837	0.8861
Anshik, V Gela and S Madgi [18]	0.8819	0.7964	0.837
D Kim, S-Y Shin, H-W Lim and S Kim [19]	0.7932	0.8393	0.8156
Our System	0.8285	0.8698	0.8486

Anshik, V Gela and S Madgi [18] extracted features such as PoS information, disease terms listed in the MeSH ontology and the word cluster generated from the word2vec model trained on Google News within a context window of two and employed the linear chain CRF model to deal with the FHIE subtask. They also depended on rules to detect the family side attributes. D Kim, S-Y Shin, H-W Lim and S Kim [19] compiled dictionaries for each type of the concepts and recognized them by a pattern matching method. They seem to employ the pattern matching method with context information to determine the family side attribute of the recognized family members. In addition, to overcome the low coverage issue, they specifically extended the dictionary for the clinical observations by collecting disease, symptom, and drug names from the Mayo Clinic website.

It is noteworthy that all of the top-ranked teams depended on rule-based approaches to determine the family side information. This study presented a unique work considering to incorporate the side and relative information in the tag scheme design. Table 6 summarizes the methods and resources used by the top-ranked teams. To investigate the practicality of these resources, we further conducted experiments to estimate the performance of our proposed architecture with the different word embeddings used by other teams. Specifically, based on the same hyper-parameters given in Table 3, we replaced the original pre-trained word embedding layer of our best run with the randomly initialized 200 dimensional word vector (denoted as Rand), the GloVe (denoted as Glove) representation and the word2vec vector trained with Google News (denoted as GN). These word vectors were non-static and will be fine-tuned during training. We also studied the performance of our model by freezing the weight of the original pre-trained vector (denoted as Freezed), and by using the pre-trained vector released by B Chiu, G Crichton, A Korhonen and S Pyysalo [13] but trained with a narrow context window size of two (Win2).

**Table 6:** Summary of The Methods and Resource Used by All Participating Teams in the Family History Information Extraction Task

Type	Description
Methodology	CRF, Bidirectional LSTM-CRF, Bidirectional CNN-LSTM-CRF, Pattern
Word embedding	GloVe: https://nlp.stanford.edu/projects/glove/ word2vec: https://code.google.com/archive/p/word2vec/ https://github.com/cambridgeltl/BioNLP-2016
Part-of-speech	NLTK (Natural language toolkit), MedPost
Ontology/Lexicon	MeSH, Mayo Clinic website and UMLS embedding (https://github.com/clinicalml/embeddings)

**Fig. 5:**
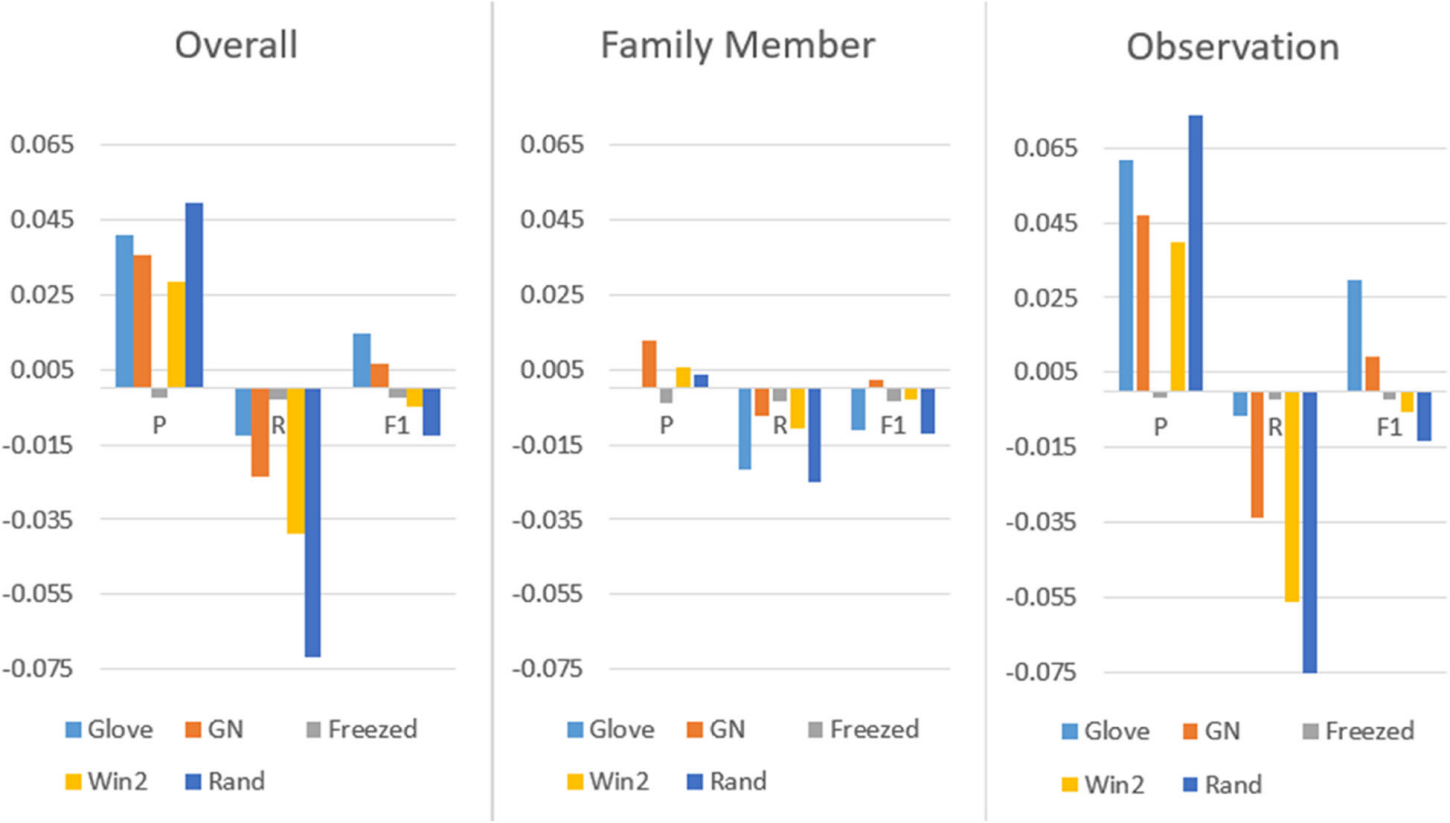
Performance comparison by using different word embeddings

Figure 5 outlines the results of the comparative evaluation in accordance with our best run. The results can be categorized into 2 different groups. The first has demonstrated improved F-scores such as Glove and GN, while the second including Freezed, Win2 and Rand acquired lower F-scores in comparison to our best run. We also noticed that the models in the first group exhibited enhanced precision for the observation concept, which contributed to an increase in the overall performance. However, they also obtained lower recalls than our best run for the family member concept. The Glove model demonstrated the best overall performance, with an improvement in precision and F-score of 0.041 and 0.015 when compared to our original model, respectively.

As expected, the randomly initialized model attained the lowest overall F-score, which seems to overfit the training set because only words that appeared in the training set were tuned. The models with the static embedding and Win2 also suffered from the lower PRF-scores on both family member and observation concept types. Although whether to fine tune the word embedding or not when using pre-trained embedding remains to be discussed [20–22], our best implementation in which a dropout was added for the non-static pre-trained word embedding demonstrated a better performance in the FHIE task. On the other hand, the lower F-score of the model with Win2 contradicts the observation of B Chiu, G Crichton, A Korhonen and S Pyysalo [13], in which they stated that the embedding with a narrow window is more suitable than larger context window for tasks like entity recognition. One possible explanation is that the evaluation of the FHIE subtask involves factors beyond simply modelling the functional similarity among words.

### Impact of the UMLS embedding features

**Fig. 6:**
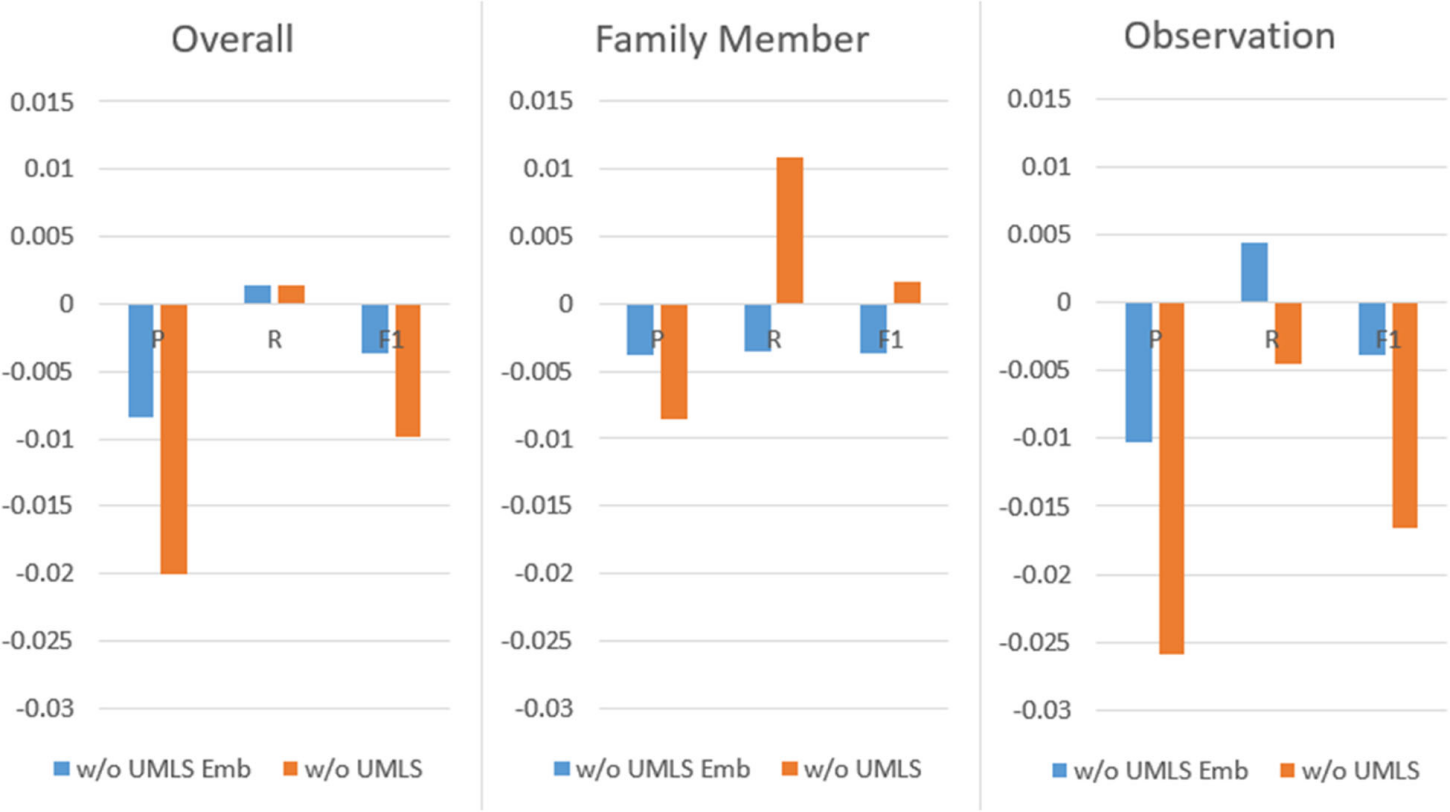
Performance comparison without the UMLS (embedding) features

Based on the same hyper-parameters, Fig. 6 illustrates the comparison between the performance of our best run and that of the models without UMLS embedding and without the entire UMLS feature on the test set. We can see that the inclusion of the feature can improve the model’s ability to recognize the observation concepts, as the removal of this feature lead to more FPs and FNs for the observation concepts. Analyses of the recognition results indicate that with the UMLS features, the model can help recognize observations unseen in the training set like “unspecified background retinopathy (CUI: C0004608)”. On the other hand, because UMLS also contains a semantic type definition for family members (“famg” in Table 2), inclusion of the features also impacts the recognition of the family member concept by increasing the precision at the expense of the recall.

### Pros and cons of the proposed tagging schemes and error cases

**Fig. 7:**
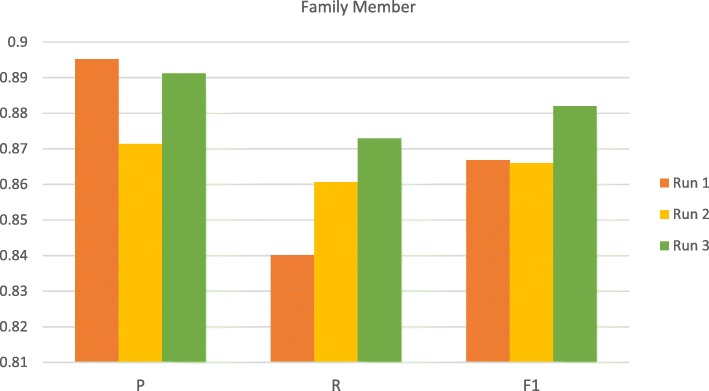
The performance comparison of the three submitted runs without considering the family side attributes

We observed that the normalization process for the *fm* property is relatively easy as not too many term variations in describing family members were encountered when developing the normalization rules simply based on the training set. Figure 7 shows the performance comparison on the test set without considering the family side attributes. Under this configuration, we can notice that the model with the standard scheme acquired the best recall and F-score, while the relation-side scheme had the worst precision and F-score. Due to the small size of the FHIE corpus, application of the standard tag scheme resulted in more training instances for each family member. By contrast, employing the relation-side scheme leads to less and imbalanced training instances. This explains the reason why the inclusion of the relation information did not improve the model’s ability in recognizing family member information based on the current annotations of the FHIE corpus.

Alternatively, it was noted that the implementation of the relation-side scheme enables the model to distinguish second-degree relatives from first-degree relatives, which is a major issue suffered by the other two tag schemes in which they failed to normalize descriptions related to the family members of someone else other than the patient. For example, the note may include the family members of the patient’s husband like his father, his mother, or other companions of the patient’s family members. By contrast, the model with the relation-side scheme can successfully identify the Aunt relationship in descriptions like “her mother had five additional [**sisters**]” in the training set. However, it may also lead to an incorrect identification of the Aunt relationship in the description “… her paternal grandmother has seven **sisters** …” that appeared in the test set. Therefore, we believe that if the size of the corpus can be enlarged, the power of the relation-side scheme may be unleashed.

On the other hand, the determination of the *sf* property is more difficult. The rule-based approach developed for the baseline configuration achieved the lowest F1-score of 0.7928 on the recognition of family members, which was lower than the best configuration by 0.061, demonstrates the advantage of encoding the side information in the tag scheme. Regarding the test set, we noticed that some errors of the baseline configuration can be solved by extending the original rules developed based on the training set by considering the context following the recognized members. Take the two sentences “Two **cousins** on the **maternal** side of his family died of myocardial infarction at ages 39 and 36” and “The patient also has one male and one female **cousin** on the **maternal** side; both are healthy” as examples. The side attribute of the mention “cousin” can be identified by looking for the maternal keyword after it. Nevertheless, extension of rules may also lead to more FP cases if there are more than one relatives mentioned in the same sentence.

The *sf* property can be hard to distinguish if cross-sentence inference is required. For instance, in the following paragraph excerpted from the training set, correct perception of the family side attribute of the member as paternal demands the knowledge to connect the term “his” to the patient’s father.

The patient’s father died at age 89 of some unknown type of asthma. **A sister**_**[paternal]**_ of his has been diagnosed with schizophrenia in her 40s.

We would like to employ intra- and inter-sentence attention mechanisms to learn to focus on specific parts of the input sentence [23] and digest cross-sentence information [24, 25] to address the above issues.

Finally, in order to understand the most challenging family member descriptions that appeared in the test set, we aggregated the results of our three submitted runs and compared the results with the gold standard annotations of the test set to investigate the most difficult cases which all three models failed to identify. The cases uncovered are listed in Table 7. The first case displayed in Table 7 is one of the most challenging FP cases as the names of family members (e.g. uncle, aunt) were not directly indicated in the text. Instead, they were indirectly referred to by outlining their relations with other members. Some FN cases were caused by the limited training instances in the training set. For example, the family member “parent” only had two training instances, and the term “sibship” in the sixth case in Table 7 has never appeared in the training set.

**Table 7:** The challenging cases in the test set of the family information extraction entity recognition subtask. The family mentions in italic and bold face were false positive cases

1	Leah’s father’s brother_[uncle/paternal]_, a 35-year-old gentleman, is considered by .. Leah’s father’s_[grandfather/paternal]_ 33-year-old sister_[aunt/paternal]_ is described as dysmorphic with dysmorphic and ... Leah’s father’s mother_[grandmother/paternal]_ developed unilateral renal artery stenosis … That lady’s sister_[aunt/paternal]_ is reported to have coronary artery disease …
2	Suzanne has a maternal aunt who died at age 55 of a liver cancer, and this aunt has **two healthy sons**_**[cousin/maternal]**_.
3	One of **her father’s brothers**_**[uncle/paternal]**_ died 1 week after birth. The cause is unknown. One of his **siblings**_**[uncle/paternal]**_ was alcoholic. …
4	Ms. Natividad’s father is healthy at the age of 80. He had one sibling, ***a sister***_**[aunt/paternal]**_, who died
5	Mrs. Manuela reports a maternal aunt had **a child**_**[cousin/maternal]**_ with heart disease.
6	The father died at age 89 with hydrocephalus. In his **sibship**_**[sibling/maternal]**_, there is late onset pelvic cancer, heart disease, and **one sister**_**[aunt/maternal**]_ had cysticercosis throughout her adult life of unknown cause.
7	Her mother did have a total of five healthy **children**_**[sibling/maternal]**_.
8	The patient’s next sister was diagnosed with schizophrenia at the age of 43. … She has **a daughter** and **a son** who are both in their 30s.
9	Suzanne’s husband is 20 and has autism. His ***brother*** died at age 66 of strokes and was thought to have depression.
10	Hannelore has a healthy 38-year-old sister who is a carrier for urethral cancer and has a healthy 7-month-old ***daughter***.
11	The father’s ***maternal grandmother*** died in her 20s of cystic fibrosis carrier.

Some annotation errors in the corpus were also revealed during the error analysis. For instance, the gold standard annotations for the family side attributes for case number 2, 4, 6 and 7 in Table 7 are “NA”. In addition, similar to the observation on the training set, the FP cases include family member mentions that require cross-sentence inferences (e.g. case number 8 and 9) or the resolution of co-references (e.g. case number 10). We also noticed that our models were confused with the description containing the mention of paternal/maternal like case number 11, which is actually the paternal great grandmother of the patient.

### Effectiveness of the CRF-based inference layer

**Fig. 8:**
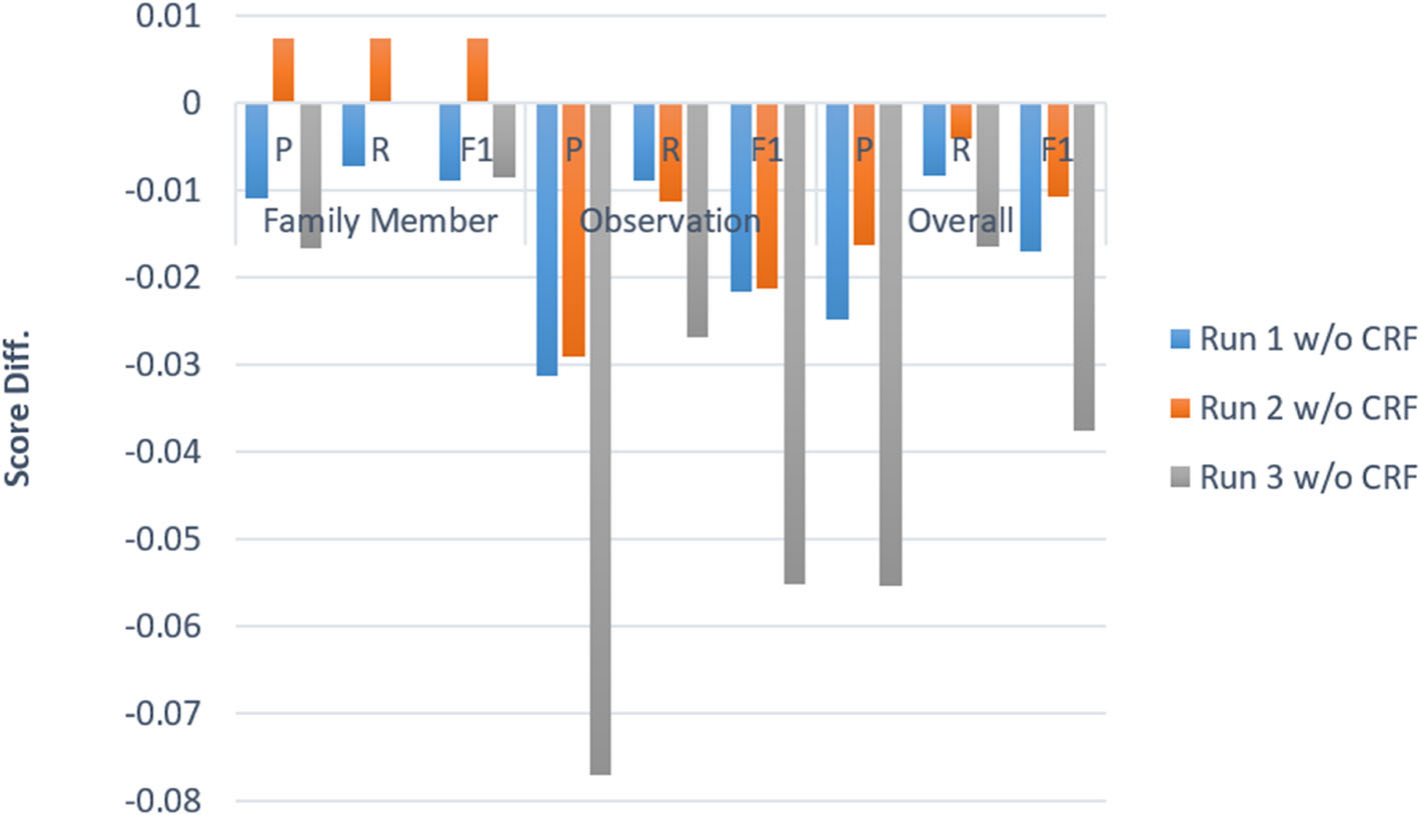
Performance of the submitted runs without adding the CRF inference layer

For the task of sequential labeling, there are dependencies between labels assigned to the tokens in a sentence. As a result, several sequential labeling studies have proposed to apply CRF as the last layer to consider the transition between labels and jointly decode the best chain of labels for a given input sentence [12, 26–28]. Figure 8 compares the performance of the models with and without adding the layer for the task of FHIE. We can see that the overall F-scores of all three configurations decreased if the CRF layer was removed. For the family member concepts, the PRF-scores of all models without CRF layers were lower than that with the layers with only one exception ─ the “Run 2 w/o CRF”. It is worth noting that the PRF-scores of the model for recognizing family members are improved by ~ 0.007 where the F-score is even better than that of “Run 1 w/o CRF”. This may be owing to the fact that the family member concepts described by using one word only occupy 62.0 and 64.8% of the training and test sets, revealing the benefit of encoding the *fm* property under such circumstance. In particular, we noticed that the model is able to successfully normalize the family member concepts described below which cannot be resolved by using current rules along with the side tag scheme.

“Her first four pregnancies were through a previous partner. The first three of these resulted in full-term [female_**Daughter**_], full-term [female_**Daughter**_], and full-term [male_**Son**_] …”.

## Conclusions

In this study, we have developed systems for FHIE based on the FHIE corpus released by the BioCreative/OHNLP challenge. We explored three tag schemes specifically designed for family member recognition and side attribute assignment. Under the current size of the released dataset, we observed that the use of side scheme along with the proposed neural network architecture and post-processing rules performed best for the FHI recognition subtask. Although the model with the relation-side scheme exhibited a lower F-score, it has the potential to distinguish second-degree relatives from first-degree relatives if the size of the corpus can be enlarged. Regarding the normalization process, we noticed that the normalization of the side family attribute is more difficult than that of the family member. Error analysis revealed challenges like cross-sentence and intra-sentence inferences which need to be investigated hereafter. Future works include the tuning of hyper-parameters, an in-depth study of the application of different methods and sizes of word embeddings, understanding the impact of fine-tuning of the pre-trained vectors, and the inclusion of the attention model to address the recognized challenges.

## Data Availability

The dataset used and analyzed during the current study are available by contacting the organizers of the BioCreative/OHNLP Challenge 2018.

## Abbreviations

CNN: Convolutional neural network
CRF: Conditional random field
CUI: Concept unique identifier
CV: Cross validation
EHR: Electronic health record
FHI: Family history information
FHIE: Family history information extraction
FN: False negative
FP: False positive
Globe Vector: GloVe
LSTM: Long short-term memory
NLP: Natural language processing
PoS: Part-of-speech
RNN: Recurrent neural network
TP: True positive
UMLS: Unified Medical Language System
